# Quantification and characterization of lung fibrosis in ARDS patients using picrosirius red staining

**DOI:** 10.3389/fmed.2025.1726436

**Published:** 2026-01-12

**Authors:** Ludovic Gerard, Marylene Lecocq, Guillaume Courtoy, Caroline Bouzin, Delphine Hoton, Joao Pinto Pereira, Thomas Planté-Bordeneuve, Antoine Froidure, Valérie Lacroix, Charles Pilette

**Affiliations:** 1 Department of Critical Care Medicine, Cliniques Universitaires Saint-Luc, Université catholique de Louvain (UCLouvain), Brussels, Belgium; 2 Pôle de Pneumologie, O.R.L. et Dermatologie (LuNS, Lung-Nose-Skin), Institut de Recherche Expérimentale et Clinique, Université catholique de Louvain (UCLouvain), Brussels, Belgium; 3 Laboratory of Experimental Pathology (EXPA), Vrije Universiteit Brussel, Brussels, Belgium; 4 Department of Pathology, Universitair Ziekenhuis Brussel, Brussels, Belgium; 5 IREC Imaging Platform, Institut de Recherche Expérimentale et Clinique, Université catholique de Louvain (UCLouvain), Brussels, Belgium; 6 Department of Pathology, Cliniques Universitaires Saint-Luc, Université catholique de Louvain (UCLouvain), Brussels, Belgium; 7 Department of Pulmonology, CHU-UCL Namur, Yvoir, Belgium; 8 Department of Pulmonology, Cliniques Universitaires Saint-Luc, Université catholique de Louvain (UCLouvain), Brussels, Belgium; 9 Department of Cardiovascular and Thoracic Surgery, Cliniques Universitaires Saint Luc, Université catholique de Louvain (UCLouvain), Brussels, Belgium; 10 Institution Université Catholique de Louvain (UCLouvain), Cliniques Universitaires Saint-Luc, Brussels, Belgium

**Keywords:** ARDS, collagen, extra-cellular matrix, fibrosis, picrosirius red

## Abstract

**Introduction:**

Evolution toward diffuse lung fibrosis is common in patients with ARDS but remains poorly characterized. In particular, the quantification and characterization of collagen fibers in lung tissue from patients with ARDS has not been performed and remain technically challenging. This study aims to precisely quantify and characterize collagen deposition in lung tissue from patients with ARDS.

**Materials and methods:**

Lung samples from ARDS patients and controls were retrospectively analyzed to quantify lung tissue fibrosis using computer-assisted digital processing after picrosirius red (PSR) staining of whole tissue sections. Additional analyses were conducted using immunofluorescence (IF) in conjunction with PSR staining and using examination of PSR stained sections under polarized light (PL).

**Results:**

Compared to controls, patients with ARDS exhibited increased total collagen deposition (*p* = 0.0006). Morphological segmentation of the collagen fibers and subsequent classification based on their staining intensity identified a subtype of fibers, termed “scattered” fibers, whose deposition was significantly increased in lung tissue of patients with ARDS compared to controls (*p* < 0.0001). The extent of scattered fibers was independent of ARDS etiology, histology or duration, but was correlated with impaired lung mechanics, impeded gas exchange, and adverse clinical outcomes. The PSR-based distinction between scattered and compact collagen fibers did not seem to reflect differences in the relative amounts of type I and type III collagen. However, the two fiber types were associated with distinct characteristics observable under polarized light microscopy.

**Conclusion:**

Lung fibrosis during ARDS is predominantly driven by the deposition of weakly stained, loose segments of collagen fibers, which is associated with impaired lung mechanics and poorer clinical outcomes. Future studies should define candidates for early anti-fibrotic therapies.

## Introduction

Acute Respiratory Distress Syndrome (ARDS) is a common cause of respiratory failure in critically ill patients (1), characterized by inflammation-mediated alveolar/capillary barrier dysfunction with interstitial and airspace edema, resulting in severe hypoxemia (2). While most patients achieve full recovery, others will progress toward extensive lung fibrosis, associated with significant mortality and prolonged mechanical ventilation (3–5). The factors determining the evolution toward restoration of normal pulmonary architecture or irreversible lung fibrosis are poorly understood, but involve a crosstalk between multiple alveolar cell types, immune and mesenchymal cells and components of the extracellular matrix (ECM) (2, 6). However, precise quantification and characterization of lung ECM, while of critical importance to improve our understanding of the mechanisms underlying fibrosis and tissue repair after acute lung injury (7), remain challenging. Most scoring methods used so far are applied on random fields instead of whole slide scans, provide only semi-quantitative evaluation, require advanced expertise in histopathology and present considerable inter- and intra-observer variability (8). Recently, novel imaging and quantification techniques for analyzing the pulmonary ECM have emerged (9). Among them, a computer assisted method for quantifying and characterizing collagen fibers in whole tissue sections stained with picrosirius red (PSR) was recently validated in experimental models of various organs, including the lungs (10). Picrosirius red (PSR) staining offers numerous advantages: it is a simple, fast, cost-effective technique, with high binding affinity for fibrillary collagen (11). Moreover, it provides consistent and high-contrast staining, allowing reliable fibrosis evaluation with less variability compared with Masson’s trichrome staining (12, 13).

This study aims to provide a precise quantification and characterization of collagen deposition in patients with ARDS and to evaluate whether fibrosis characteristics are associated with disease severity, lung mechanics or clinical outcomes. To address these objectives, we conducted a retrospective analysis of lung tissue samples of patients with ARDS and controls subjects. Lung tissue fibrosis was characterized and quantified using computer-assisted digital processing after picrosirius red (PSR) staining of whole tissue sections.

## Materials and methods

Additional details regarding patient selection, data collection, immunostaining protocols and imaging technique are available in the online data supplement.

### Patients

Patients with ARDS were screened within a database of all patients who underwent open-lung biopsy for non-resolving ARDS or for ARDS of unknown etiology (2007–2021, flow chart in Supplementary Figure 1). The control group comprised lung tissue samples from patients with a normal lung function who underwent lobectomy for a solitary lung tumor, with sections taken at maximal distance from the lesions. Patients and controls characteristics are summarized in Table 1.

**TABLE 1:** Baseline characteristics, adjunctive treatments, tissue sampling characteristics and outcomes in the 12 controls and 49 patients with ARDS.

Variables	Controls (*n* = 12)	ARDS (*n* = 49)	*P*
Age, years	57.5 (36–68)	61 (53–76)	0.08
Male, n (%)	7 (58)	30 (61.2)	0.51
Body mass index, kg/m^2^	24.2 (21.4–27.2)	23.5 (20.9–27.8)	0.86
Immunosuppression, n (%)	2 (16)	24 (49)	0.020
Diabetes, n (%)	1 (8)	6 (12.2)	0.55
Active smoking, n (%)	5 (42)	10 (20.5)	0.12
Chronic obstructive pulmonary disease, n (%)	0	2 (4.1)	0.67
Other chronic respiratory disease, n (%)	1 (8)	3 (6.1)	0.56
Active neoplasia, n (%)	12 (100)	12 (24.5)	<0.001
APACHE II (ARDS diagnosis)		18 (15–23)	
SOFA (ARDS diagnosis)		6 (4–9)	
PaO_2_/FiO_2_ (ARDS diagnosis), mmHg		133 (53)	
**Cause of ARDS, n (%)**			
COVID-19		0	
Pneumonia		19 (38.8)	
Sepsis (of extra-pulmonary origin)		6 (12.2)	
Other		24 (48.9)	
**Adjunctive treatments during ICU stay, n (%)**			
Prone positioning		19 (38.7)	
Inhaled nitric oxide		16 (32.6)	
NMBA		4 (8.2)	
ECMO		1 (2)	
Vasopressors		27 (55.1)	
Renal replacement therapy		16 (32.6)	
SOFA (OLB day)		7 (5–9)	
PaO_2_/FiO_2_ (OLB), mmHg (*n* = 46)		127 (108–161)	
PaCO_2_ (OLB), mmHg		46 (42–56)	
PEEP (OLB), cmH_2_O		10 (7–11)	
VT (OLB), ml/kg PBW		7.1 (6.3–7.9)	
Pplat (OLB), cmH_2_O		25 (21–30)	
Compliance (OLB), mL/cmH_2_O		26.5 (19.1–43.3)	
**ARDS severity (OLB), n (%) (*n* = 46)**			
Mild		8 (17.4)	
Moderate		35 (76)	
Severe		3 (6.5)	
Duration of ARDS before tissue sampling, days		7 (2.5–13.5)	
Diffuse alveolar damage on pathological examination, n (%)		25 (51)	
28-day mortality n (%)		21 (42.8)	
VFDays D28, days		5 (1–12)	
ICU LOS, days		23 (13–39)	

ARDS, acute respiratory distress syndrome; SOFA, Sequential Organ Failure Assessment; APACHE II, Acute Physiology and Chronic Health Evaluation II; NMBA, Neuromuscular Blocking Agents; ECMO, Extra-Corporeal Membrane Oxygenation; OLB, Open Lung Biopsy; PEEP, Positive End-Expiratory Pressure; VT, Volume Tidal; Pplat, plateau Pressure; VFDays D28, Ventilator Free Days at day 28; ICU, Intensive Care Unit; LOS, Length of Stay. Data are median (IQR).

### Open lung biopsy

OLB was conducted in the event of persistent respiratory failure, after ongoing lung infection was ruled out [via bronchoalveolar lavage (BAL) or via endotracheal aspirates], or when an alternative diagnosis was suspected, based on patient history, clinical and radiological presentation. In most cases, OLB was performed at patient bedside in the ICU. In all cases, lung biopsy was performed using mini-thoracotomy. For each patient, one large lung sample was taken from an area chosen based on the distribution of the lesions on a lung CT-scan and on surgical accessibility.

### Lung tissue immunohistochemistry and imaging

#### Picrosirius red staining, imaging, and quantification

**FIGURE 1:**
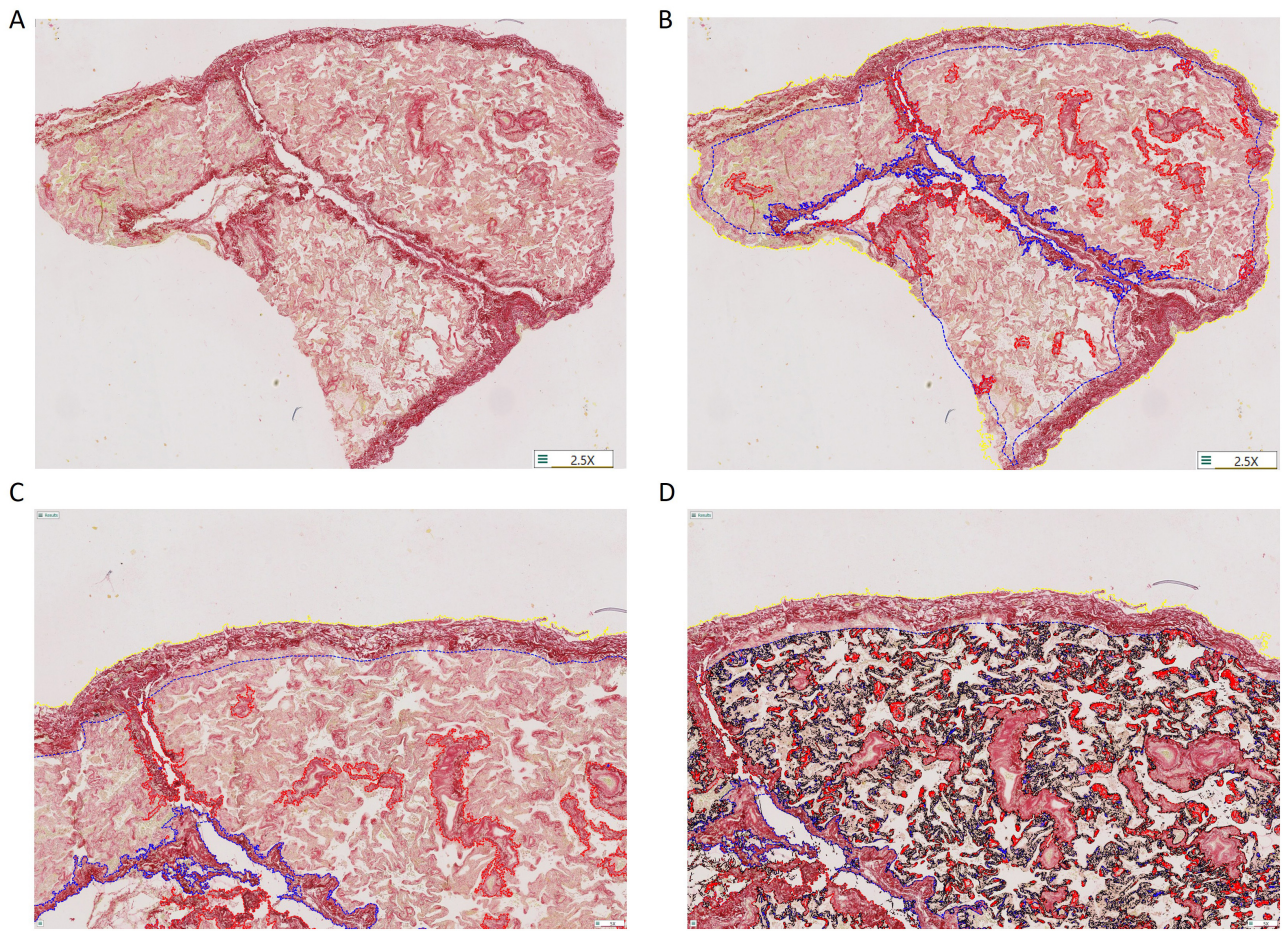
Description of the methodology used for semi-automated quantitative evaluation of lung tissue fibrosis in whole tissue sections stained with picrosirius red (PSR) using the image analysis tool Author version 2023.01 (Visiopharm, Hørsholm, Denmark). **(A)** Representative lung tissue section stained with picrosirius red. **(B,C)** Manual delineation of the parenchymal area, as opposed to peri-bronchial/peri-vascular and pleural areas (within red and blue ticked lines, respectively). **(D)** Automated detection of collagen fibers based on the intensity of PSR staining. Magnification 2.5x **(A,B)** or 5x **(C,D)**.

Fibrosis was quantified using picrosirius red (PSR) stained sections as described previously (10). One section per patient was analyzed. Whole tissue sections were digitalized using an Axioscan.z1 slide scanner (Zeiss, Germany) at x20 magnification. For each slide series, acquisition parameters, including LED power and exposure times were kept constant across all slides. No background subtraction was applied. Stainings were then quantified on entire tissue sections with software applications (“APP”s) using the image analysis tool Author version 2023.01 (Visiopharm, Hørsholm, Denmark). As shown in Figure 1, within the tissue, large bronchi and vessels (>0.2 mm diameter) were excluded from the analysis (Figure 1B, higher magnification in Figure 1C), because they were surrounded by large connective tissue, and its presence was highly variable between sections, constituting a bias. Pleural areas, which were highly heterogenous in terms of thickness and extension between samples, were included in a separate analysis. In the remaining tissue, which was considered as lung parenchyma and constituted the region of interest (ROI), PSR staining was detected after optimization of the signal for increased selectivity and specificity (Figure 1D) (10). PSR staining was quantified as the sum of positive areas detected in all the ROIs of the whole tissue section, out of the area analyzed (total area of the ROI) and referred to as collagen proportional area (CPA, expressed in %). The formula used to calculate CPA can be expressed as follows:


C⁢P⁢A=∑stained⁢area⁢within⁢selected⁢ROIarea⁢total⁢ROI⁢x⁢ 100⁢(i⁢n%)

Furthermore, after optimized detection, PSR-stained collagen fibers were subsequently segmented by watershed and separate objects software functions, a method previously validated in multiple organs (10). Segments were then classified according to their intensity values into strongly stained segments (compact fibers) or weakly stained segments (scattered fibers) and further quantified as the area occupied by each fiber type out of the area analyzed (expressed in %). All sections were stained, digitalized and quantified in a single batch, with the same parameters. As shown in Supplementary Figure 2, we performed three different analyses, manually adjusting the threshold parameters to classify fibers as “scattered” or “compact” (Supplementary Figures 2B–D) with consistent results (Supplementary Figures 2E–G). We selected one of these set of parameters (Supplementary Figures 2D,G) for subsequent analysis based on its enhanced ability to segregate controls from patients with ARDS.

#### Duplex fluorescence immunostaining in lung tissue and superposition with PSR

Formalin-fixed, paraffin-embedded (FFPE) tissue processing and tyramide signal amplification (TSA)-based fluorescence immunostaining were performed as previously described (14). Sections were stained immunohistochemically with collagen I α1 chain (Col1a1), and with collagen III α1 chain (Col3a1). The list of reagents used can be found in Supplementary Table 1. Duplex immunostained slides were digitalized in fluorescence using an Axioscan.z1 (Zeiss, Germany) slide scanner at x20 magnification. After coverslip removal and washings, sections were stained with PSR and scanned again with the same slide scanner. Both scans were carefully aligned using the tissue alignment tool of Visiopharm software. Detection and classification of the collagen fibers was performed on the PSR staining. Compact and scattered areas detected in PSR were defined as the regions of interest (ROI). The mean fluorescence intensity (MFI) for each fluorescent channel of the superimposed fluorescent scan was then calculated as the average pixel intensity within each ROI (the sum of intensities in each pixel of the ROI, divided by the total number of pixels in the ROI), after background substraction (from an unstained region). Fluorescence intensities were reported in arbritraty units (AU).

#### Polarized light microscopy

Collagen fiber density was quantified on tissue sections stained with Picro-Sirius Red and imaged by polarized-light microscopy using a Zeiss Axioscan.Z1 equipped with a x20 objective. As previously described (15), birefringent color hues reflect collagen fiber thickness and packing, with green indicating thin or loosely packed fibers and orange to red indicating progressively thicker and/or more tightly packed fibers.

Image analysis was performed using QuPath (16). A supervised classifier was trained to identify green, orange, and red birefringent collagen signals. The classifier was applied to manually delineated parenchyma and pleura regions, with bronchi and vessels excluded from the analysis.

### Statistical analysis

Statistical analyses were performed using SPSS 21 (IBM, Armonk, NY) and figures were created using GraphPad Prism 10 (GraphPad Software, LaJolla, CA). Values were expressed as median [interquartile range (IQR)] unless otherwise stated. Categorical and continuous variables were analysed using the chi-square test and Mann-Whitney test, respectively. Correlation between continuous variables was analysed using Pearson’s correlation test. Furthermore, survival curves were generated using the Kaplan-Meier method, compared by log-rank test. The optimal cut-off point of CPA between the two survival groups was calculated with a receiver operating characteristic (ROC) analysis. All tests were two-sided, with a significance level set at 0.05.

## Results

### Increase in total and scattered collagen fibers in patients with ARDS

**FIGURE 2:**
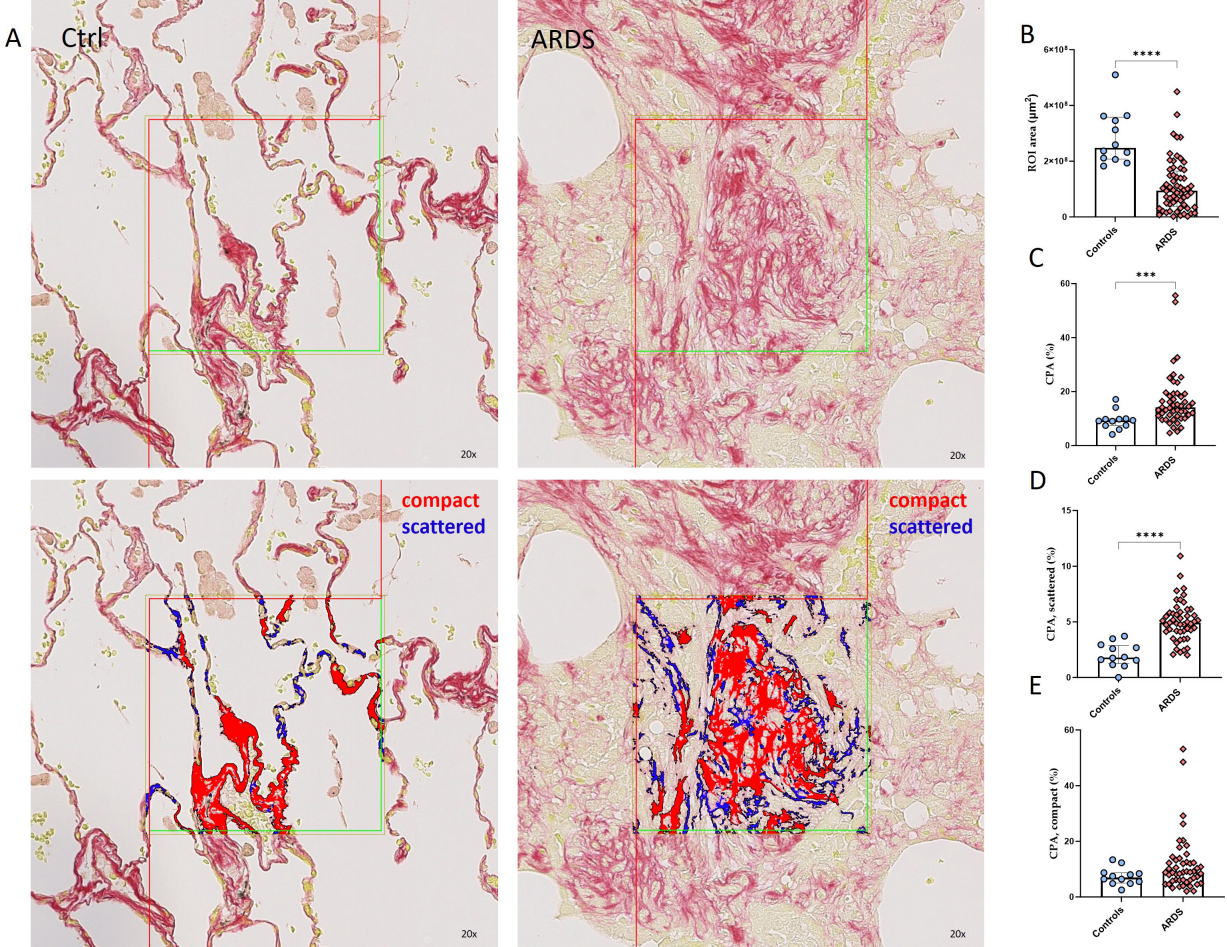
Increase in total and scattered collagen fibers in patients with ARDS. **(A)** Representative picrosirius red (PSR) staining of a lung tissue section of control (left) and a patient with ARDS (right) before (upper panel) and after (lower panel) automated detection and segmentation of lung tissue fibrosis, between compact fibers (red) and scattered fibers (blue). Magnification 20x. **(B)** Comparison of the Region of Interest (ROI) area between controls and ARDS, showing that the samples from controls (*n* = 12) were significantly larger than samples from ARDS patients (*n* = 49). **(C–E)** PSR staining was quantified as the sum of positive areas detected in all the ROIs of the whole tissue section (after exclusion of large bronchiae, vessels and pleurae), out of the area analyzed (total area of the ROI) and referred to as collagen proportional area (CPA, expressed in %). Quantification of the CPA of all fibers **(C)**, scattered fibers **(D)** and compact fibers **(E)**, showing an increase in CPA of total and scattered fibers in patients with ARDS (*n* = 49) compared to controls (*n* = 12). Each dot represents one patient. Between group difference was evaluated using the non-parametric Mann-Whitney U test. ***p < 0.0005, ****p < 0.0001.

Lung tissue from patients with ARDS (*n* = 49) and controls (*n* = 12) (baseline and detailed characteristics available in Table 1) was stained with PSR, then digitalized and processed for quantitative evaluation of collagen deposition, expressed as collagen proportional area (CPA) (Figure 2A). The dimensions of the samples were significantly higher for controls (lobectomies) than for ARDS patients (open-lung biopsy). The median size of the samples was 247.9 × 10^6^ μm^2^ (IQR 207.4 × 10^6^–357.7 × 10^6^) in controls vs. 94.7 × 10^6^ μm^2^ (IQR 37 ×10^6^–164.3 × 10^6^) in patients with ARDS (*p* < 0.0001) (Figure 2B). To better characterize lung fibrosis, collagen fibers were then segmented into “compact” fibers (strongly stained, dense segments) and “scattered” fibers (weakly stained, loose segments) based on density and staining intensity (Figure 2A). Total CPA was significantly higher in patients with ARDS compared to controls (Figure 2C), [CPA of 14.2 % (IQR 11.0–19.2), vs. 9.7 % (7.5–9.9), *p* = 0.0006], indicating increased collagen deposition. Patients with ARDS exhibited a dramatic increase in scattered fibers compared to controls (Figure 2D), [CPA of 4.9% (4.1–5.8) vs. 1.8 (1.3–3.7), *p* < 0.0001], while the increase in compact fibers (Figure 2E), [CPA 8.9 % (5.9–12.9) vs. 7.0 (5.0–8.7), *p* = 0.16] was modest, and did not reach statistical significance. Additionally, there was no difference in the CPA of total or scattered fibers based on the etiology of ARDS (Supplementary Figures 3A,B) or based on the histologic pattern observed in lung tissue biopsies (Supplementary Figures 3C,D). Similarly, no correlation was found between CPA (for total or scattered fibers) and duration of ARDS before lung tissue sampling (Supplementary Figures 4A,B).

### The extent of scattered fibers in lung tissue of patients with ARDS correlates with impaired lung mechanics and increase in arterial partial pressure in CO_2_ (PaCO_2_)

**FIGURE 3:**
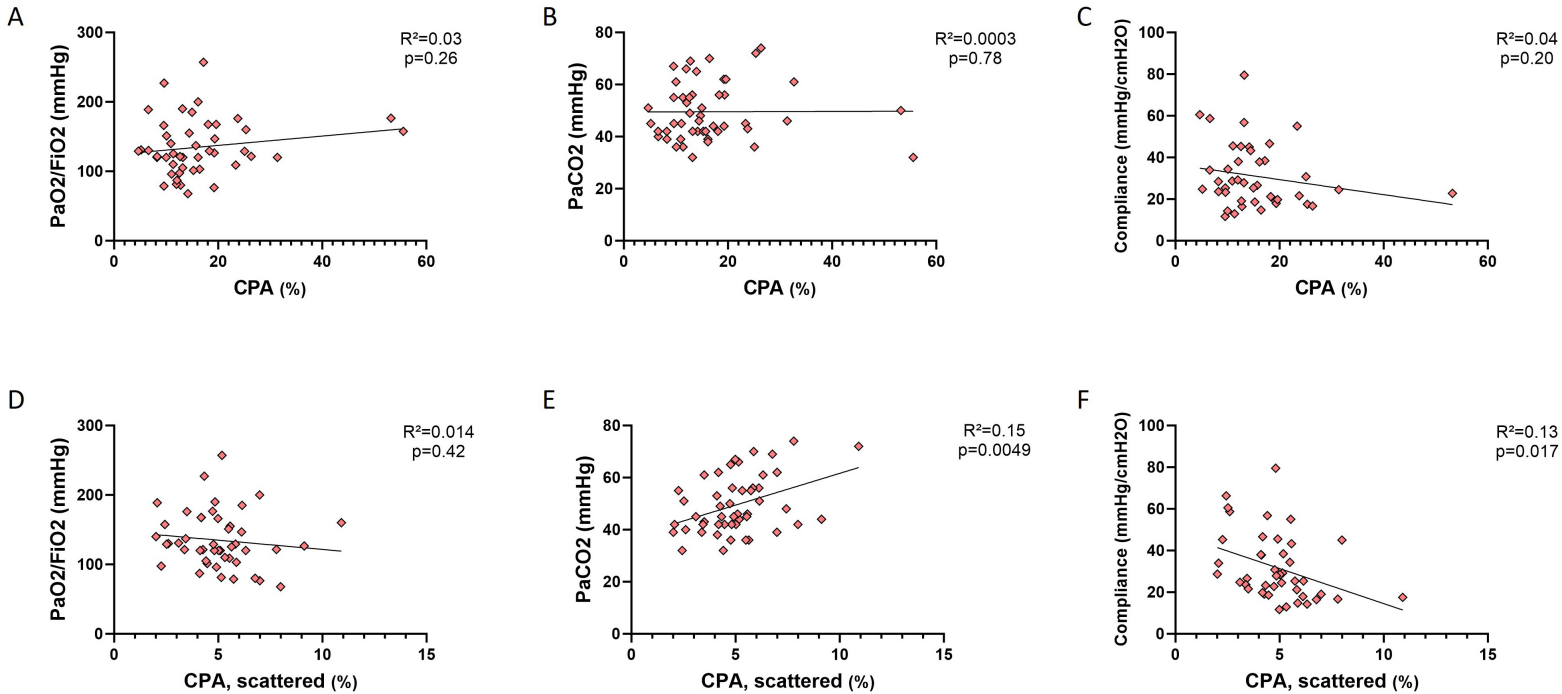
Correlation between automated quantification of collagen proportional area, ARDS severity and lung mechanics. Pearson’s tests of the correlation between CPA of all collagen fibers (upper panel) or CPA of scattered fibers (lower panel) with PaO_2_/FiO_2_ ratio **(A,D)**, PaCO_2_ **(B,E)** and static respiratory system (RS) compliance **(C,F)**, showing a positive correlation between CPA of scattered fibers and PaCO_2_ (**E**, *R* = 0.39, *p* = 0.0049), and a negative correlation between CPA of scattered fibers and static RS compliance (**F**, *R* = –0.36, *p* = 0.017).

We next investigated whether collagen deposition correlated with ARDS severity, with PaCO_2_ or with mechanical ventilation (MV) parameters at the time of OLB. All patients were ventilated in volume control ventilation, and measures were recorded immediately before surgical lung biopsy. As shown in Figure 3, there was no significant correlation between total CPA and ARDS severity or mechanical ventilation parameters, measured right before open-lung biopsy (Figures 3A–C). In contrast, a significant correlation was observed between the CPA of scattered fibers and PaCO_2_ (Figure 3E) (*R* = 0.39, *p* = 0.0049), as well as static lung compliance (calculated as tidal volume divided by driving pressure, Figure 3F, *R* = −0.36, *p* = 0.017).

### The increase in scattered fibers is associated with deleterious clinical outcomes in patients with ARDS

**FIGURE 4:**
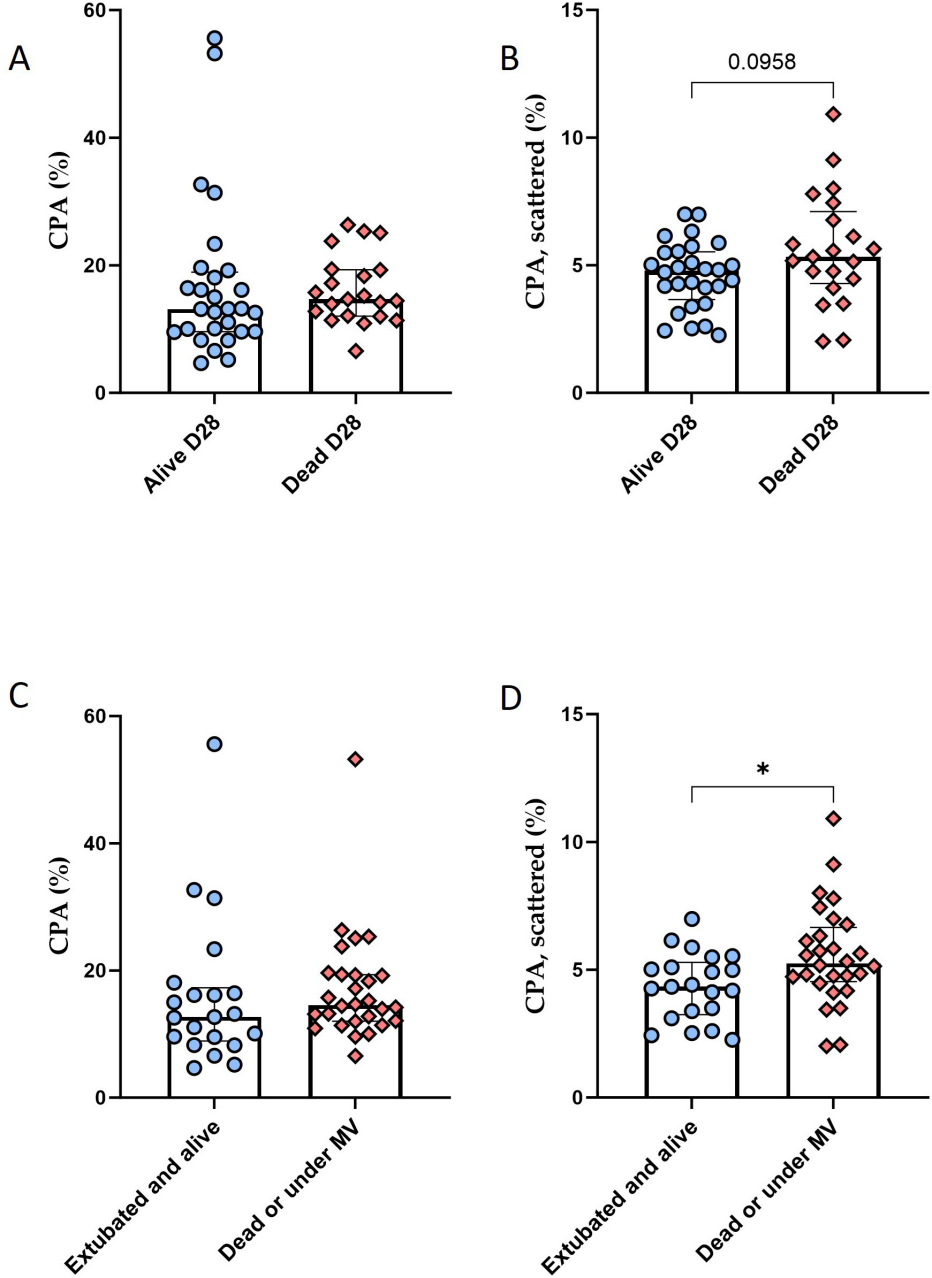
Decreased CPA of scattered fibers in patients with favorable clinical outcomes. **(A,B)** Comparison of CPA of all collagen fibers **(A)** or CPA of scattered fibers **(B)** between patients who survived up to day 28, as compared to the patients who died before day 28, showing a trend toward higher CPA of scattered fibers in patients who survived after day 28 (*p* = 0.096). **(C,D)** Comparison of CPA of all collagen fibers **(C)** or of scattered fibers **(D)** in patients who were successfully weaned off mechanical ventilation at day 28, compared to patients who were either dead or still under MV at day 28, showing a lower CPA of scattered fibers in patients successfully weaned off MV (*p* = 0.026). Between group difference was evaluated using the non-parametric Mann-Whitney U test. *p < 0.05.

As shown in Figure 4, no significant association was found between total CPA and all-cause mortality at 28 days, whereas there was a non-significant trend toward higher scattered CPA at the time of OLB in patients who died within 28 days following OLB (*p* = 0.098). Notably, patients who were extubated and alive at day 28 after OLB (*n* = 21) had a significantly lower CPA for scattered fibers at the time of OLB (Figure 4D) [4.3% (3.2–5.3) vs. 5.3% [(4.5–6.7), *p* = 0.026] when compared to patients who were either dead or still under mechanical ventilation (MV) (*n* = 28). To further explore the association between fibrosis and outcomes, patients were classified into a “high fibrosis” or a “low fibrosis” group, based on their CPA for scattered fibers. The cut-off value defining whether a patient is at risk of mortality in the 28 days following lung biopsy with the highest sensitivity and specificity was found at 5,11% [ROC area under the curve (AUC) = 0.64, sensitivity = 0.62, specificity = 0.71]. It was therefore defined as the threshold between the “high fibrosis” and the “low fibrosis” groups for Kaplan-Meier analysis. The main characteristics of these groups can be found in Table 2.

**TABLE 2:** Baseline characteristics, adjunctive treatments, tissue sampling characteristics and outcomes in 49 patients with ARDS, separated according to their respective values of “CPA scattered” (<5.11% vs. >5.11%).

Variables	Low CPA scattered (*n* = 28)	High CPA scattered (*n* = 21)	*P*
Age, years	61 (54–77)	64 (50.5–76.5)	0.80
Male, n (%)	17 (60.7)	13 (61.9)	0.59
Body mass index (kg/m^2^)	23.3 (20.9–26.5)	23.4 (19.1–26.7)	0.79
Diabetes, n (%)	4 (14.2)	2 (9.5)	0.46
Active smoking, n (%)	7 (25)	3 (14)	0.27
Chronic obstructive pulmonary disease, n (%)	2 (7.1)	0	0.31
Other chronic respiratory disease, n (%)	3 (10.7)	0	0.17
Active neoplasia, n (%)	8 (28.6)	4 (19)	0.31
Immunosuppression	13 (46.4)	11 (52.4)	0.61
APACHE II	17 (15–24)	19.5 (14.2–23)	0.61
SOFA (ARDS diagnosis)	6 (4–9)	5.5 (4–7)	0.52
**Cause of ARDS, n (%)**			
Pneumonia	10 (35.7)	9 (42.9)	0.35
Sepsis (of extra-pulmonary origin)	2 (7.1)	4 (19)	0.22
Other	16 (57.4)	8 (38.1)	0.12
**Adjunctive treatments, n (%)**			
Prone positioning	10 (35.7)	9 (42.9)	0.41
Inhaled Nitric oxide	7 (25)	9 (42.9)	0.16
NMBA	2 (7.1)	2 (9.5)	0.58
ECMO	0	1 (4.8)	0.43
Vasopressors	14 (50)	13 (61.9)	0.30
Renal replacement therapy	9 (32.1)	7 (33.3)	0.59
ARDS severity (OLB), n (%) (*n* = 46)			0.30
Mild	3 (11.5)	5 (25)	
Moderate	22 (84.6)	13 (65)	
Severe	1 (3.5)	2 (10)	
Diffuse alveolar damage on pathological examination, n (%)	13 (46.4)	12 (57.1)	0.32
SOFA (OLB day)	8 (4–10)	7 (5–9)	0.83
PaO_2_/FiO_2_ (OLB), mmHg (*n* = 46)	129 (120–167)	123 (87–154)	0.27
PaCO_2_ (OLB), mmHg	44 (40.5–52.5)	55 (44–64)	0.031
PEEP (OLB), cmH2O	10 (7–12)	10 (6–10)	0.46
Plateau Pressure (OLB), cmH_2_O (*n* = 45)	24 (20–29)	27 (21–33)	0.15
VT (OLB), ml/kg PBW	7.1 (6.6–7.8)	6.9 (6.3–8.0)	0.66
Compliance (OLB, mmHg/cmH_2_O)	28.6 (23.1–45.8)	21.2 (16.6–36.4)	0.039
Duration of ARDS before tissue sampling, days	6 (2–14)	7 (2–13)	0.66
ICU LOS, days	24 (15–41)	18 (10–39)	0.39
ICU mortality, n (%)	11 (39.3)	14 (66.7)	0.053
Day 28 mortality, n (%)	8 (28.6)	13 (61.9)	0.020
MV free days D28	6 (2–16)	1 (0–10)	0.026

ARDS, acute respiratory distress syndrome; SOFA, Sequential Organ Failure Assessment; APACHE II, Acute Physiology and Chronic Health Evaluation II; NMBA, Neuromuscular Blocking Agents; ECMO, Extra-Corporeal Membrane Oxygenation; OLB, Open Lung Biopsy; PEEP, Positive End-Expiratory Pressure; VT, Volume Tidal; ICU, Intensive Care Unit; LOS, Length of Stay; MV free days D28, mechanical ventilation free days at day 28. Results are median (IQR).

**FIGURE 5:**
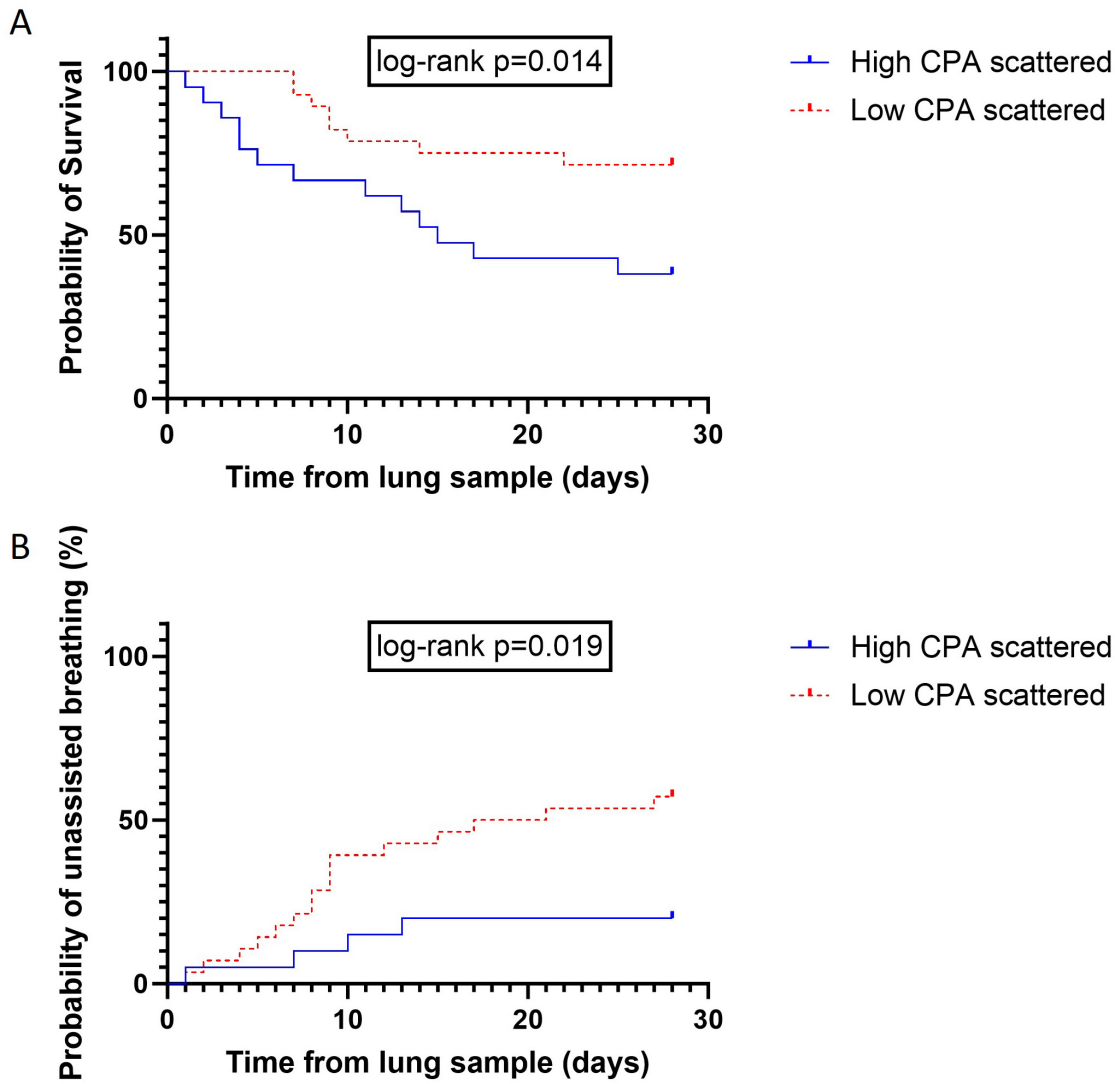
Improved clinical outcomes in patients with lower collagen proportional area of the scattered fibers. **(A)** Kaplan Meier survival curve comparing patients with a high vs. low collagen proportional area (CPA) for scattered fibers, showing increased 28 days survival in the group of patients with the lowest CPA for scattered fibers (*p* = 0.014). **(B)** Kaplan Meier survival curve comparing patients with a high vs. low CPA for scattered fibers, showing an increase in the proportion of patients breathing without assistance on day 28 in the group of patients with the lowest CPA for scattered fibers (*p* = 0.019). Between group difference was evaluated using Log-rank test.

Demographic variables, ARDS etiologies, adjuvant treatments, ARDS severity and MV settings at the time of OLB, or ARDS histology were similar between groups. Patients in the “high fibrosis” group demonstrated impaired lung mechanics, reflected by a lower static lung compliance (*p* = 0.039), impeded gas exchange, with a higher PaCO_2_ (*p* = 0.033) and adverse outcomes, with significantly fewer MV-free days at D28 (*p* = 0.026). Additionally, as shown in Figure 5, patients in the “high fibrosis” group had higher all-cause mortality (Figure 5A) (log-rank *p* = 0.014) and a lower probability of unassisted breathing (Figure 5B) (log-rank *p* = 0.019) by day 28.

### The characteristics of subpleural fibrosis differ from those of parenchymal fibrosis in patients with ARDS

**FIGURE 6:**
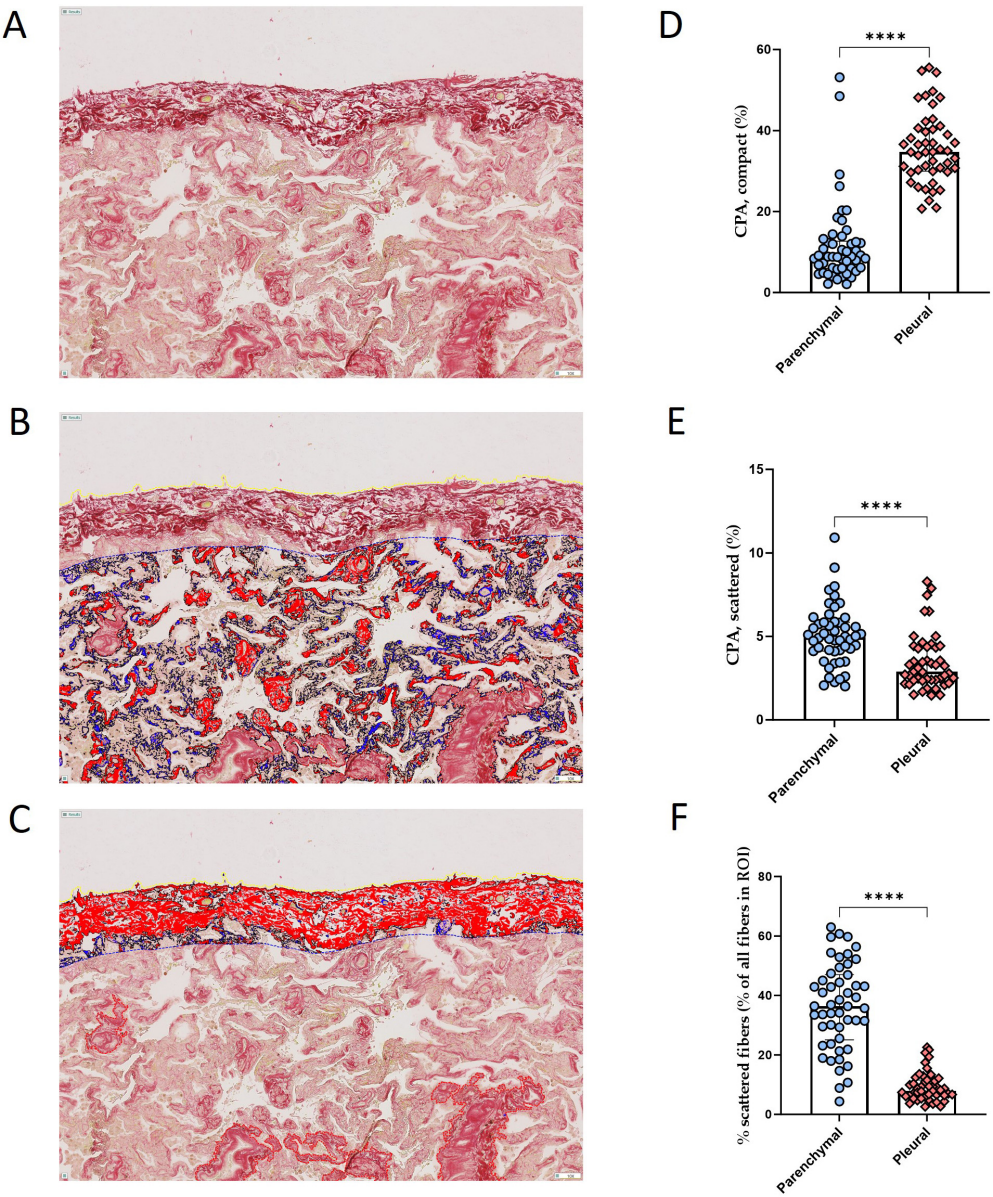
Comparison of the abundance and characteristics of subpleural fibrosis and lung parenchymal fibrosis. **(A–C)** Representative picrosirius red staining of a lung tissue section of a patient with ARDS, before **(A)** and after **(B,C)** automated detection and segmentation of tissue fibrosis between compact (red) and scattered (blue) collagen fibers in the lung parenchyma **(B)** and in the subpleural area **(C)**. Magnification 10x. **(D,E)** Quantification of the collagen proportionate area (CPA) of compact fibers **(D)** and scattered fibers **(E)**, showing a higher abundance of compact fibers and a lower abundance of scattered fibers in pleural areas compared to lung parenchyma. **(F)** Quantification of the area of scattered fibers expressed as a proportion of the total area of all detected collagen fibers, showing a greater proportion of scattered fibers in the lung parenchyma than in pleural regions. Each dot represents one patient. Between group difference was evaluated using the non-parametric Mann-Whitney U test. ****p < 0.0001.

We next sought to compare the abundance and properties of collagen fibers in the lung parenchyma and pleural regions. To do so, we performed a separate analysis involving collagen quantification and characterization in the pleural areas using the same thresholds to classify the fibers as either “compact” or “scattered.” As shown in Figure 6, subpleural fibrosis was predominantly composed of compact fibers [CPA of 34.7 % (30–40.5)] (Figures 6A–D). Conversely, scattered fibers were less abundant in pleural area than in the lung parenchyma, both in absolute terms [CPA of 2.9% (2.4–4.4) vs. 4.9 % (4.1–5.9), *p* < 0.0001] (Figure 6E) and as proportion of all collagen fibers detected in each region of interest [7.9% (6.1–11.5) vs. 36.4% (25.1–47), *p* < 0.0001] (Figure 6F).

### Collagen I and III involvement in the formation of “scattered” collagen fibers

**FIGURE 7:**
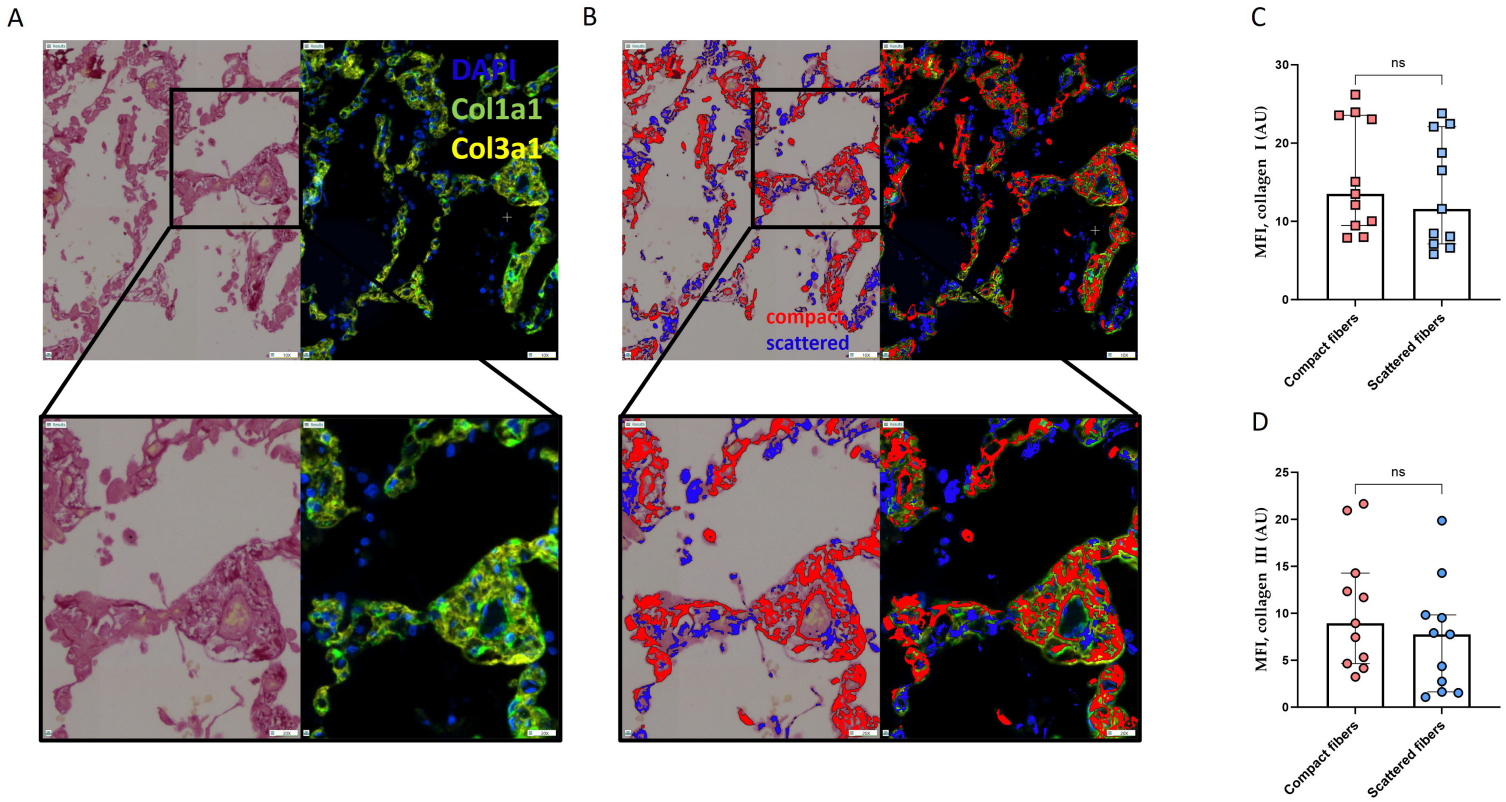
Superposition between compact and scattered fibers detected in PSR and subtypes of collagen selectively stained using immunofluorescence. **(A)** Representative picrosirius red (PSR) staining (left) and duplex immunofluorescence (IF) staining (right) for collagen I α1 chain (Col1a1, green), and with collagen III α1 chain (Col3a1, yellow) and DAPI (blue) of a representative lung tissue section of a patient with ARDS. Upper panel: magnification 10x, lower panel: magnification 20x. **(B)** Superposition of the automated detection and segmentation of lung tissue fibrosis, between compact fibers (red) and scattered fibers (blue) on IF staining. Upper panel: magnification 10x, lower panel: magnification 20x. **(C,D)** Quantification of the mean fluorescence intensity (MFI), expressed in Arbitrary Units (AU), for collagen I and collagen III on lung tissue sections stained with IF, in the areas superposed with compact and scattered fibers, in both controls and patients with ARDS. MFI of collagen I **(C)** and collagen III **(D)** did not differ between compact and scattered fibers. Between group difference was evaluated using the non-parametric Mann-Whitney U test.

To better understand the biological significance of scattered collagen fibers, we investigated their relative abundance for type I and type III collagens, the two collagen subtypes most associated with lung fibrosis in ARDS patients (17, 18). Duplex IF staining for collagen I and collagen III (Figure 7A, green and yellow, respectively) followed by PSR staining (Figure 7B) were performed on the same lung tissue sections of a randomly selected subset of patients with ARDS and controls. After precise PSR and fluorescent scans alignment, mean fluorescence intensities (MFI) for collagen I and collagen III were calculated in the area classified as compact and scattered fibers (Figures 7C,D). There was no difference in MFI neither for collagen I (Figure 7C) nor collagen III (Figure 7D) in scattered and in compact fibers, indicating that the different aspect of fibers in PSR did not arise from differences in the abundance of collagen I and III. However, MFI for collagen III was significantly higher in both compact and scattered fibers of patients with ARDS compared to controls (Supplementary Figures 5A–E). This suggests an increased abundance of collagen III in fibrotic regions of ARDS patients, regardless of fiber classification in PSR. Additionally, some areas in which collagen fibers were detected in PSR did not overlap with collagen I nor collagen III, suggesting that other subtypes of collagen fibers were also involved.

### Collagen fibers of patients with ARDS exhibit distinct characteristics in PSR stained sections observed under polarized light microscopy

**FIGURE 8:**
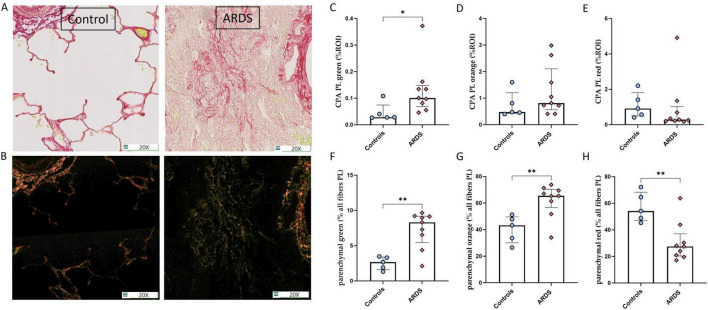
Collagen fibers of patients with ARDS exhibit distinct characteristics in PSR stained sections observed under polarized light (PL) microscopy. **(A,B)** Representative picrosirius red (PSR) staining under a brightfield microscope **(A)** and under a PL microscope **(B)** of representative lung tissue sections of a control (left) and of a patient with ARDS (right). Magnification 20x. **(C–E)** Quantification of the three different colors observed under PL microscope, expressed as the sum of positive areas detected in all the ROIs of the whole tissue section (after exclusion of large bronchiae, vessels and pleurae), out of the area analyzed (total area of the ROI) and referred to as collagen proportional area (CPA, expressed in %), showing an increase in CPA of “green” fibers **(C)** in patients with ARDS (*n* = 8) compared to controls (*n* = 5). **(F–H)** The proportion of green **(F)** and orange fibers **(G)** among all detected fibers under PL was higher, whereas red fibers **(H)** was lower in ARDS than in controls. Between group difference was evaluated using the non-parametric Mann-Whitney U test. *p < 0.05; **p < 0.005.

We performed a complementary analysis of PSR-stained sections stained with PSR, which were examined under polarized light in a subset of controls/ARDS patients randomly selected from the full cohort (Figures 8A,B). Interestingly, fibers in the green color under PL were more abundant in ARDS patients than in controls both in absolute terms [CPA of 0.1% (0.07–0.15) vs. 0.028% (0.027–0.074), *p* = 0.019] (Figure 8C) and as a proportion of all collagen fibers detected in PL [8.3% (5.5–9.2) vs. 2.7% (1.6–3.9), *p* = 0.007] (Figure 8F). In contrast, no significant difference were observed in the absolute abundance of orange and red fibers (Figures 8D,E) although they differed in proportion (Figures 8G,H). Additionally (Supplementary Figures 6A–F), we observed a very strong correlation between CPA in polarized light (PL) and CPA in PSR (*R*^2^ = 0.77, *p* < 0.0001) (Supplementary Figure 6D). Furthermore, we observed a mild correlation (albeit statistically non-significant) between CPA of green fibers in PL and CPA of scattered fibers (*R*^2^ = 0.29, *p* = 0.07), (Supplementary Figure 6E), and a moderate correlation between CPA of red fibers in PL and CPA of compact fibers in PSR (*R*^2^ = 0.52, *p* = 0.008) (Supplementary Figure 6F).

## Discussion

In this study, we used a rigorous, computer-assisted, semi-automated quantification technique applied to whole lung tissue sections obtained via open-lung biopsy in a large cohort of patients with ARDS and controls. Our findings demonstrate an increase (of around 50%) in total collagen content in ARDS lungs. Whereas increased collagen deposition is well-established in animal models of acute lung injury (7, 19–21), to the best of our knowledge, only two previous studies have compared lung fibrosis quantification in tissue sections of small cohorts of patients with ARDS and controls, yielding contrasted results (18, 22). However, differences in patient selection, tissue sampling, image processing and quantification techniques preclude direct comparison with our analysis. Conversely, several studies have demonstrated the presence of fibrosis in the lung tissue of patients with ARDS, using qualitative or semi-quantitative evaluation by pathologists (4, 23–27). These studies reported fibroproliferation in highly variable proportions of ARDS patients who underwent lung tissue sampling, reflecting the heterogeneity between ARDS patients and the challenges of dichotomizing patients into fibrotic and non-fibrotic groups based solely on qualitative assessments. This underscores the need to adopt quantitative approaches for tissue fibrosis evaluation.

We propose a new morphological classification of collagen fibers according to their compactness, based on a proprietary algorithm previously validated in the experimental setting (10). This approach enables automated identification of a specific subset of fibers, termed scattered fibers, whose deposition is strongly increased in ARDS patients. Conversely, the area occupied by compact fibers did not differ between ARDS and control lungs, suggesting that our classification technique can help differentiate ECM fibers essential for maintaining lung tissue architecture and fibers characteristic of fibrotic processes in acute lung injury. This hypothesis is further supported by the association between the area of scattered fibers on lung and impaired lung mechanics (reduced static compliance), hindered gas exchange (increased PaCO_2_), and adverse clinical outcomes. Additionally, our findings suggest that early lung fibrosis in ARDS is primarily driven by an increase in weakly stained, loose collagen fibers, whose extent appear to be independent of ARDS origin or duration. This contrasts with previous qualitative analysis of lung tissue from ARDS autopsies, which reported fibrosis progression correlated with ARDS duration (23). However, differences in fibrosis definition, quantification techniques, and patient populations could explain this discrepancy; autopsy studies involve patients who succumbed to ARDS, whereas our cohort underwent OLB for unexplained or non-resolving ARDS, representing a different clinical subset. Furthermore, we found no difference in collagen fibers quantification according to the presence/absence of diffuse alveolar damage (DAD), suggesting that fibrotic processes occur independently of the ARDS histologic patterns. Such findings may have significant implications for identifying patients suitable for treatments targeting fibroproliferation, such as corticosteroids or other specific therapies.

Moreover, while previous studies have associated the presence of histologic fibroproliferation with increased mortality (4, 5, 24), ours is the first to demonstrate an association between the precise quantification of a collagen fiber subtype (based on morphological characteristics) and clinical outcomes, such as mortality and duration of mechanical ventilation. Notably, “less invasive” fibrosis quantification tests, based on CT scanner analysis (22, 26, 28, 29) or on biomarkers in the bronchoalveolar lavage fluid (24), have shown correlations with clinical outcomes. However, none of these methods has been rigorously validated against quantitative evaluation of lung tissue fibrosis, limiting their diagnostic reliability and applicability. Pulmonary levels of N-terminal peptide of type III procollagen (NT-PCPIII), a marker of collagen synthesis (30), have been associated with clinical outcomes (24–26, 31–34) and response to corticosteroids (35). NT-PCPIII is therefore suggested as a surrogate for the presence of fibroproliferation during ARDS, but this is based solely on qualitative evaluation of lung tissue fibrosis (24, 36). Although their invasiveness will prevent the widespread use of tissue-based quantification techniques in clinical routine, they could serve as “gold standard” to develop new or refine existing biomarker-based or CT-based fibrosis quantification tests. These non-invasive methods, once optimized, could facilitate better patient selection for treatments targeting fibroproliferation, whose beneficial effects in patients with acute respiratory failure have been recently suggested (37, 38).

Lastly, if our morphological classification allows us to detect different types of fibers based on their morphological characteristics, the question remains as to their biological significance. Previous studies have shown that collagen I and collagen III mainly drive fibrotic processes during ARDS (17, 18), findings confirmed by our IF analysis. However, PSR-based collagen fiber classification in scattered and compact fibers appears to be unrelated to differences in the relative abundance of type I and type III collagen fibers. Therefore, we hypothesize that differences in fiber staining intensity may be related to different stages of collagen fibrils polymerization rather than differences in their composition, with scattered fibers representing a newly formed state of collagen fibers (39). This hypothesis is further supported by the differences in the characteristics of the collagen fibers observed under PL, with an increase in green fibers in ARDS patients (which were mildly correlated with scattered fibers in PSR), indicating thinner and loosely packed collagen fibers. Alternatively, other ECM components, such as collagen IV or collagen VI, may contribute to these morphological differences (18, 40, 41). Besides, it was recently suggested that dynamic changes in ECM proteins took place during ARDS, with an increase in some collagen fibers such as collagen IV in later stages of ARDS (41).

Several limitations to our study must be acknowledged. First, it is a retrospective analysis, involving a single referral center, potentially impacting its generalizability. Second, lung tissue analysis was performed in patients who underwent lung biopsy, typically those with ARDS of unknown origin, or unresolving ARDS. It constitutes a highly selected subgroup of ARDS patients, with an expected high prevalence of fibrosis. Therefore, our results may not be representative of a general, unselected ARDS population and should be interpreted with caution. Third, because only one specimen per patient was analyzed, its capacity to accurately reflect the global fibrotic processes occurring across both lungs is uncertain. Fourth, because of the difficulty of accurately delineating airways and blood vessels in lungs with heavily distorted architecture, we were unable to perform a separate analysis to quantify and characterize collagen fibers surrounding the airways and blood vessels, which were already shown to differ from parenchymal collagen fibers (17, 39, 42, 43) and which may represent a significant site of pathology (42). Fifth, the technique of quantification used, which relies on computer-assisted evaluation of the intensity of tinctorial staining on digitalized slides, has been shown effective in demonstrating differences between groups, when staining can be undertaken in a tightly controlled, single-batch manner by a trained laboratory (44), which limits its applicability outside the research setting (45). However, PSR staining protocol can be standardized and the algorithms used for detecting and segmenting collagen fibers can be shared, facilitating the widespread adoption of this technique. Sixth, the characterization of collagen fibers, based on tinctorial affinity, is purely morphological, and provide little mechanistic insight, even though IF superposition analysis suggests no major change in the composition of the most common collagen fibers. Therefore, a more complete view of ECM alterations during ARDS and of the associated signaling pathways should be obtained.

Overall, this study demonstrates an increase in total collagen content in the lungs of patients with ARDS using a novel, robust and computer-assisted quantification technique applied to whole tissue sections. Additionally, we identified a subtype of collagen fibers named “scattered” fibers, characterized by their lower density and weaker staining intensity, which are significantly and selectively increased in ARDS lungs, irrespectively of ARDS etiology, duration, severity or histological aspect. Furthermore, their extent correlates with impaired lung mechanics, compromised gas exchange and deleterious clinical outcomes. Our findings underscore the potential of quantitative fibrosis assessments to advance our understanding of ECM remodeling in ARDS and highlight the need for further research into non-invasive diagnostic tools to identify ARDS patients who are candidates for targeted therapeutic strategies.

## Data Availability

The raw data supporting the conclusions of this article will be made available by the authors, without undue reservation.
