# Supplementary material for: Quantification and characterization of lung fibrosis in ARDS patients using picrosirius red staining
Source: Front Med (Lausanne). 2026 Jan 12;12:1726436. doi: 10.3389/fmed.2025.1726436 (PMC12834131; doi:10.3389/fmed.2025.1726436)
Supplement: Supplementary file 1 [file Data_Sheet_1.pdf]

**Automated quantification and characterization of lung tissue fibrosis in ARDS patients using picrosirius red staining**

Ludovic Gerard, MD PhD[1,2]; Marylene Lecocq[2]; Guillaume Courtoy, PhD[3,4]; Caroline Bouzin, PhD[5]; Delphine Hoton, MD[6]; Joao Pinto Pereira, MD[1,2]; Thomas Planté-Bordeneuve, MD PhD[2,7]; Antoine Froidure, MD PhD[2,8]; Valérie Lacroix, MD PhD[9]; Charles Pilette, MD PhD[2,8].

Online data supplement

MATERIAL AND METHODS

**Patient selection**

This study was conducted in accordance with the ethics committee approval (Comité d'Ethique Hospitalo-Facultaire, Cliniques universitaires Saint-Luc, UCLouvain N°2019/22jan/026).

ARDS group

Patients with ARDS were screened within a database of all patients who underwent open-lung-biopsy for ARDS, according to Berlin definition criteria (1) between 2007 and 2021. The main characteristics and outcomes of most of these ARDS patients have been previously reported (2). Lung biopsy was performed for non-resolving ARDS or for ARDS of unknown etiology. Among the 56 patients of the database for whom sufficient lung tissue could be retrieved, we excluded 7 patients for whom there were technical issues with staining or digitalization. The 49 remaining patients were included for morphological analysis.

Control group

The patients included in the control group (n=12) were selected within a database of patients who underwent lobectomy or surgical open lung-biopsy between 2018 and 2019. The main reason for lobectomy was cancer or metastasis resection (solitary lesion). Patients with known pulmonary disease such as chronic obstructive pulmonary disease and patients treated with systemic corticosteroids (>8 mg/day equivalent prednisolone) were excluded, to avoid potential confounding effects, as were patients who underwent lobectomy after October 2019 to avoid a potential asymptomatic contamination with SARS-CoV2. All patients were included in the immunohistochemistry morphological analysis.

***Data collection***

Data were retrospectively retrieved from Electronic Medical Recording (Qcare ICU, HIM, Germany). Demographic data, underlying conditions, relevant biological and respiratory parameters, and cause of ARDS were recorded. Acute Physiology and Chronic Health Evaluation II (APACHE II) and Sequential Organ Failure Assessment (SOFA) scores were

calculated at the day of ICU admission. The use of adjuvants therapies throughout the entire ICU stay was carefully recorded. Subjects were followed up until death or hospital discharge, and outcome was recorded at 28 days (after OLB) and ICU discharge. Data regarding lung tissue sampling and main pathological examination findings were recorded.

**Picrosirius red staining**

Five µm paraffin sections were deparaffinized, rehydrated and immersed in 1.0% phosphomolybdic acid solution for 2 min. After rinsing with water, sections were incubated in a saturated aqueous picric acid solution containing 0.1% Direct red 80 (Sigma-Aldrich, USA) #365548) for 2 hours at room temperature, then vigorously washed 2 min in 0.01 N HCL before an additional wash in water. Finally, slides were dehydrated and mounted with a Sakura automated coverslipper.

**Immunofluorescence**

Lung biopsies were fixed for 24h at 4°C in 4% formaldehyde, embedded in paraffin and sectioned. After deparaffinization, 5 µm tissue sections were processed according to the protocol described by Aboubakar et al (3). After deparaffinization in toluene and methanol, endogenous peroxidases were inhibited for 15 min by adding Bloxall (Vector Laboratories, Peterborough, UK) followed by 30 min treatment with 0.3% hydrogen peroxide. Sections were then submitted to antigen retrieval in 10 mM citrate pH 6.0 buffer containing 0.1% triton (5 min, 15 psi) and to blocking of specific antigen-binding sites (Tris buffered saline (TBS) containing 5% normal goat serum and 0.1% Tween 20). The first primary antibody was incubated in TBS containing 5% normal goat serum and 0.1% Tween 20 and detected by corresponding horseradish peroxidase (HRP)-conjugated polymer secondary antibodies for 40 min at room temperature (RT). HRP was then visualized by tyramide signal amplification (TSA) using AlexaFluor-conjugated tyramides (Thermo Fisher Scientific, Paisley, UK). After a new citrate buffer incubation step (this step also detaches antibodies to the tissue section), the same protocol was applied with other primary antibodies and different AlexaFluor-conjugated tyramides. Details regarding the antibodies used and their concentration can be found in table E1. After a washing step in PBS, nuclei were finally stained with Hoechst 33342 (Thermo Fisher Scientific) diluted in TBS containing 10% BSA and 0.1% Tween 20, washed in distilled water, and mounted with Dako fluorescence mounting medium (Agilent). Slides were stored at −20 °C until whole slide digitalization.

<u>Imaging and quantitative evaluation of immunostaining in whole tissue sections</u>
Stained slides were digitalized using an Axioscan.z1. For each slide series, acquisition parameters, including LED power and exposure times were kept constant across all slides. No background subtraction was applied. Stainings were quantified on entire tissue sections with software applications ("APP"s) using the image analysis tool Author version 2023.01 (Visiopharm, Hørsholm, Denmark). Thresholds for each fluorescent channel were manually adjusted at high magnification (x20) on representative stained regions, compared to negatively stained areas. These thresholds were then uniformly applied to all slides within the series.

| Antigen | Primary antibody | | | Secondary antibody | Fluorophore |
|---|---|---|---|---|---|
| | Dilution | company | Catalog number | Polymer HRP | Alexia Fluor |
| Collagen I alpha-1 chain | 1/1500 | Abcam | Ab138492 | rabbit | AF488 |
| Collagen III alpha-1 chain | 1/1000 | Cell signaling | 63034 | rabbit | AF555 |

**Table E1. Reagents used for duplex fluorescence immunohistochemistry.**

All analyses were performed by an experienced observer, who was blinded to the clinical categories of the tissue samples.

FIGURES

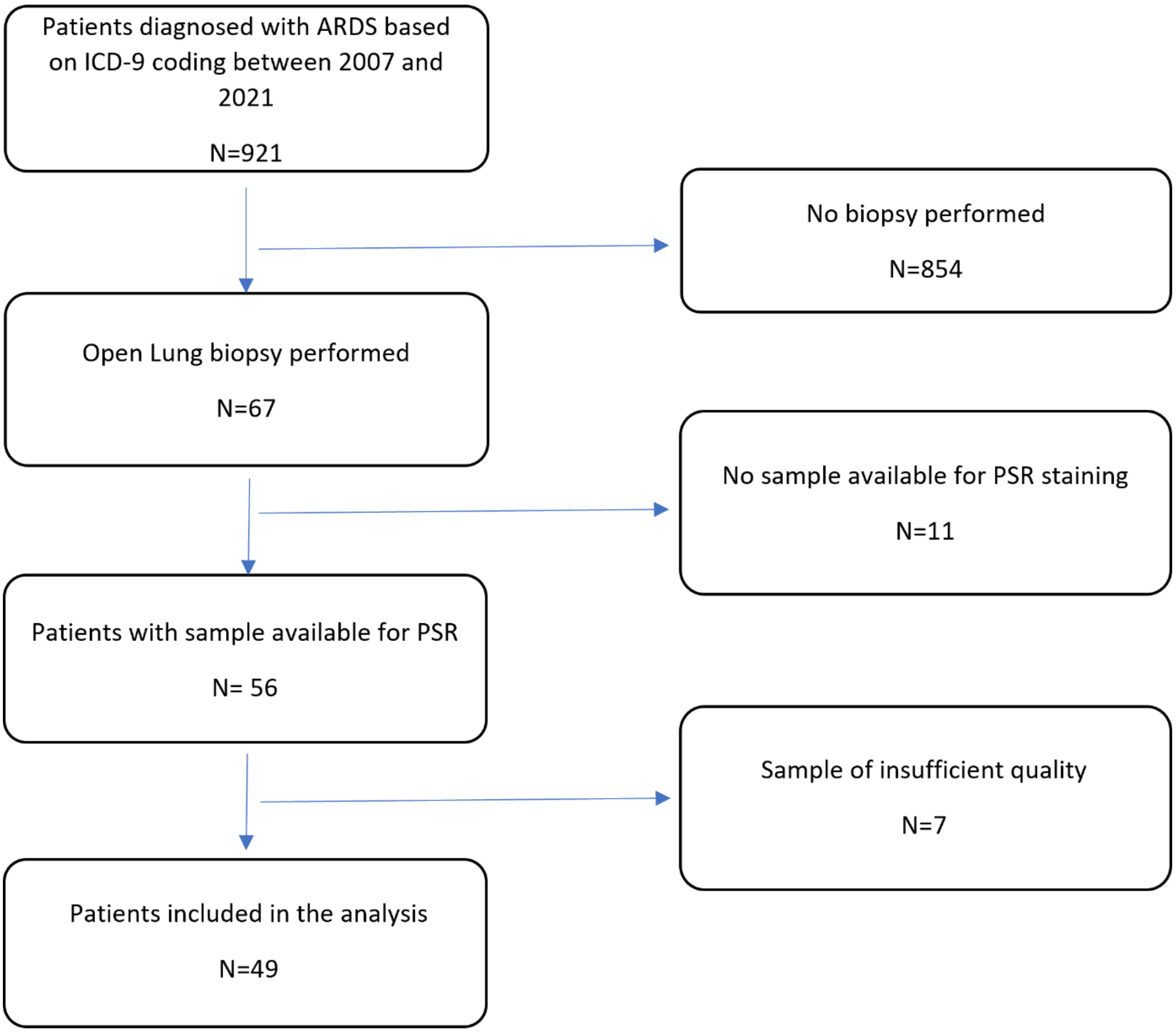


Figure S1

**Flow chart** of the patients included in the retrospective analysis of automated quantification of lung tissue fibrosis in whole tissue sections stained with picrosirius red.

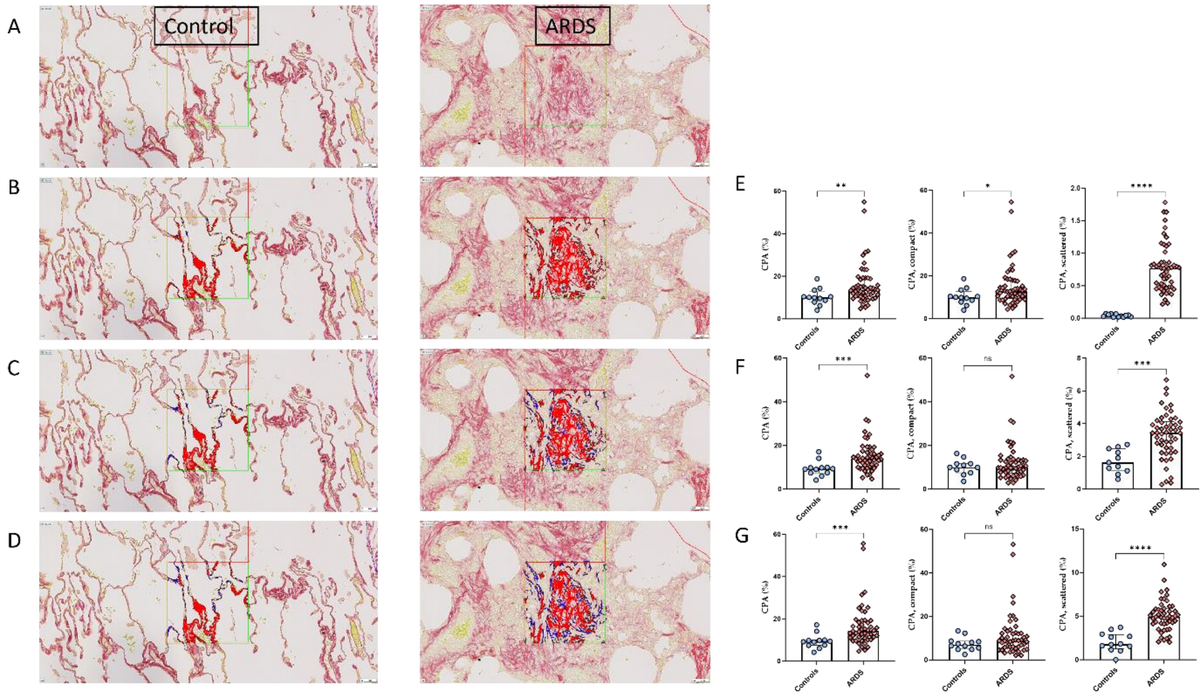


Figure S2

A-D. Representative picrosirius red staining of a control (left panel) vs a patient with ARDS (right panel), showing three different tresholds in the detection of scattered (blue) vs compact fibers (red) within the central square, ranging from the most restrictive threshold for scattered fibers (B) to the most sensitive threshold (D). E,F,G. Quantification of the collagen proportionate area (CPA) of all fibers (left panel), compact fibers (middle) and scattered fibers (right) using the three different tresholds for the detection of scattered fibers, from the most restrictive (E) to the most sensitive (G), between controls (n=12) and patients with ARDS (n=49). Between group difference was evaluated using the non-parametric Mann-Whitney U test. * indicates $p<0.05$, ** indicates $p<0.005$, *** $p<0.0005$, **** $p<0.0001$.

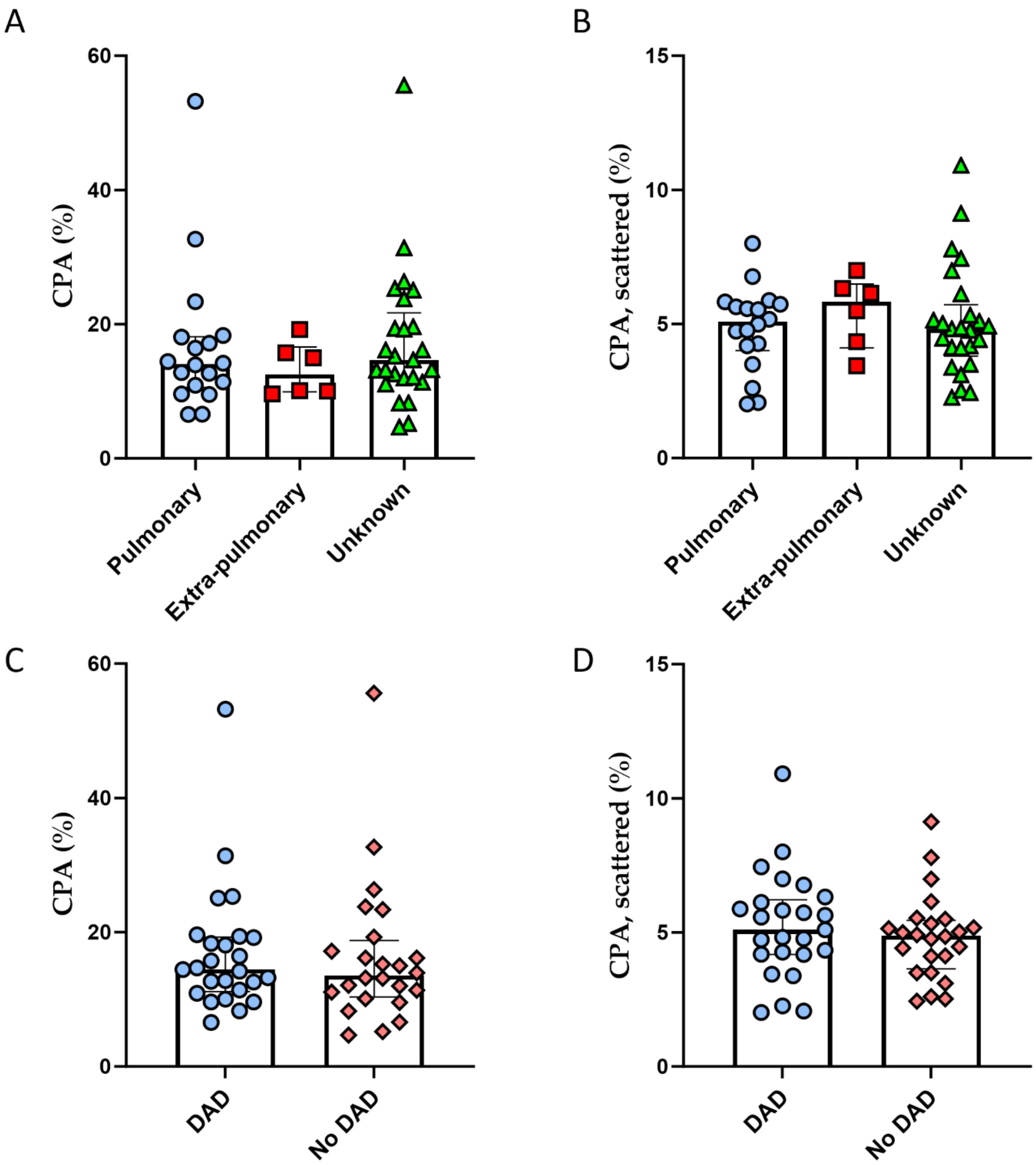


Figure S3

A,B. Comparison of CPA of all collagen fibers (A,C) or CPA of scattered fibers (B,D) according to ARDS etiology (A,B; pulmonary vs extra-pulmonary vs unknown) and according to the histological pattern shown using lung biopsy (C,D diffuse alveolar damage (DAD) vs other).

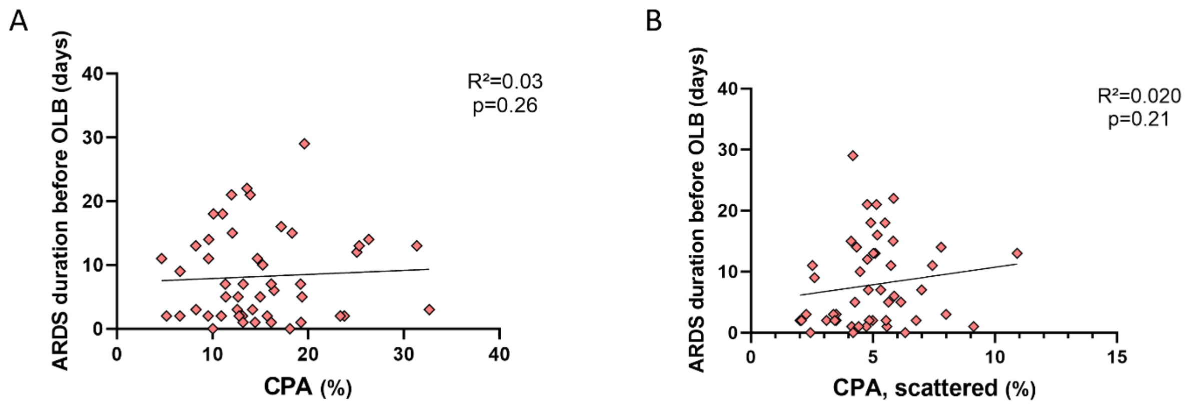


Figure S4

Correlation between automated quantification of collagen proportionate area and ARDS duration. Correlation between CPA of all collagen fibers (A) or CPA of scattered fibers (B) with time elapsed between ARDS diagnosis and OLB was studied using Pearson correlation test, showing no significant correlation between CPA or CPA of scattered fibers and timing (R=0.03, p=0.26 and R=0.02, p=0.21, respectively).

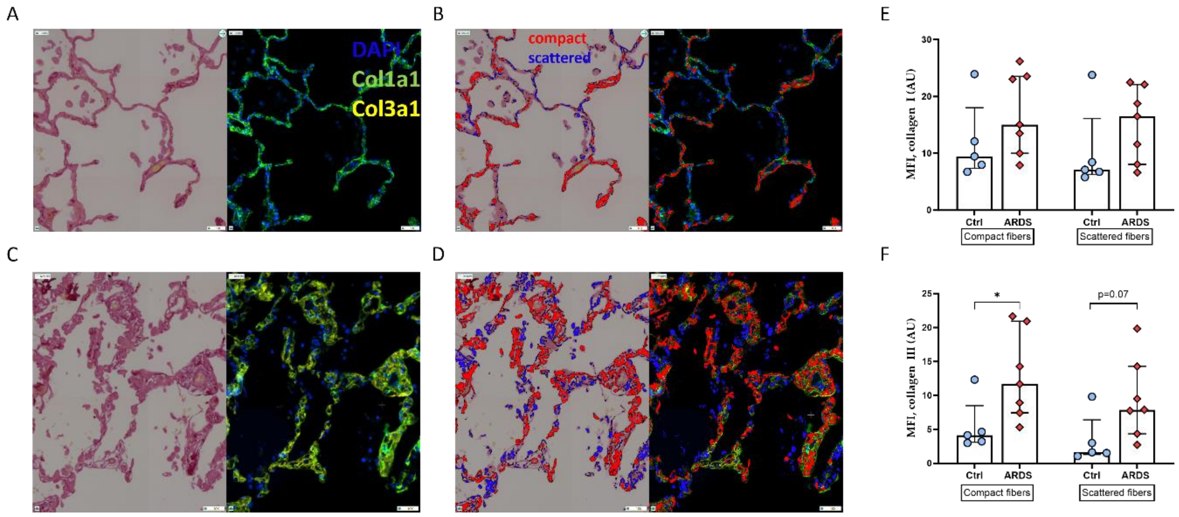


Figure S5.

Comparison of the mean fluorescence intensity of collagen I and collagen III between controls and patients with ARDS, in both compact and scattered fibers. A,C. Representative picrosirius red (PSR) staining (left) and duplex immunofluorescence (IF) staining (right) for collagen I α1 chain (Col1a1, green), and with collagen III α1 chain (Col3a1, yellow) and DAPI (blue) of a representative lung tissue section of a control (A) and a patient with ARDS (C). B,D. Superposition of the automated detection and segmentation of lung tissue fibrosis, between compact fibers (red) and scattered fibers (blue) on lung tissue section of the same control (B) and ARDS patient (D). Magnification 10x. Detection was performed in PSR, then was superposed on IF stained section after careful tissue alignment. E-G. Quantification of the mean fluorescence intensity (MFI) for collagen I (E) and collagen III (F) on lung tissues sections stained with IF, in the areas superposed with compact and scattered fibers, between controls (n=5) and patients with ARDS (n=7). AU stands for Arbitrary Unit. Between group difference was evaluated using the non-parametric Mann-Whitney U test. *indicates $p<0.05$.

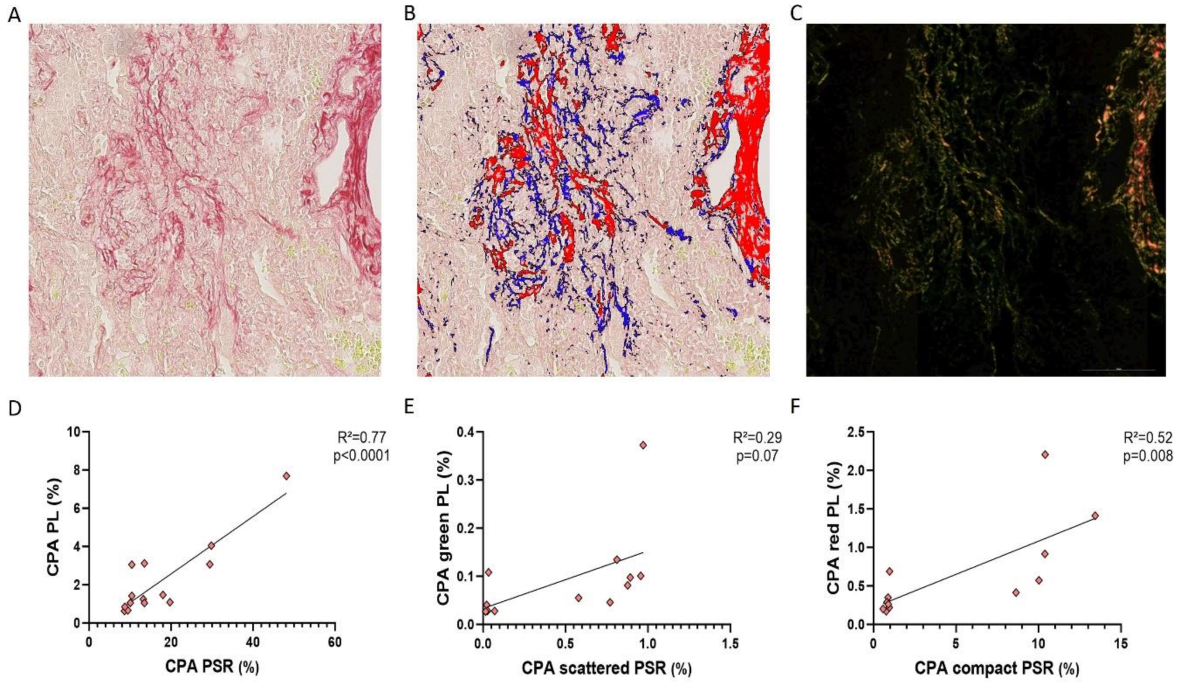


Figure S6. A-B. Representative picrosirius red (PSR) staining viewed under a brightfield microscope, shown before (A) and after (B) automated detection and segmentation of lung tissue fibrosis, distinguishing compact fibers (red) and scattered fibers (blue) of a representative lung tissue section of a patient with ARDS. C. The same tissue section stained with PSR and examined under polarized light microscopy. D-F. Correlations between automated quantification of collagen proportionate area (CPA) in PSR and PL: total collagen fibers (D), scattered collagen fibers in PSR and green fibers in PL (E) and compact fibers in PSR and red fibers in PL, using Pearson correlation tests.

1. Grasselli G, Calfee CS, Camporota L, Poole D, Amato MBP, Antonelli M, et al. ESICM guidelines on acute respiratory distress syndrome: definition, phenotyping and respiratory support strategies. Intensive Care Med. 2023;49(7):727-59.
2. Gerard L, Bidoul T, Castanares-Zapatero D, Wittebole X, Lacroix V, Froidure A, et al. Open Lung Biopsy in Nonresolving Acute Respiratory Distress Syndrome Commonly Identifies Corticosteroid-Sensitive Pathologies, Associated With Better Outcome. Crit Care Med. 2018;46(6):907-14.
3. Aboubakar Nana F, Hoton D, Ambroise J, Lecocq M, Vanderputten M, Sibille Y, et al. Increased Expression and Activation of FAK in Small-Cell Lung Cancer Compared to Non-Small-Cell Lung Cancer. Cancers (Basel). 2019;11(10).
